# Mental workload and neural efficiency quantified in the prefrontal cortex using fNIRS

**DOI:** 10.1038/s41598-017-05378-x

**Published:** 2017-07-12

**Authors:** Mickaël Causse, Zarrin Chua, Vsevolod Peysakhovich, Natalia Del Campo, Nadine Matton

**Affiliations:** 1 Institut Supérieur de l’Aéronautique et de l’Espace (ISAE-SUPAERO), Toulouse, France; 2 Ecole de psychologie, Université Laval, Québec, Canada; 3 Centre of Excellence in Neurodegeneration of Toulouse, NeuroToul, CHU Toulouse France; 4 Toulouse NeuroImaging Center, ToNIC, University of Toulouse, Inserm, UPS, Toulouse France; 5 University of Cambridge, Department of Psychiatry, Addenbrooke’s Hospital, Cambridge, UK; 6 Ecole Nationale de l’Aviation Civile, Toulouse, 31055 France; 7 Laboratoire CLLE-LTC, 5 Allée Antonio Machado, 31100 Toulouse, France

## Abstract

An improved understanding of how the brain allocates mental resources as a function of task difficulty is critical for enhancing human performance. Functional near infrared spectroscopy (fNIRS) is a field-deployable optical brain monitoring technology that provides a direct measure of cerebral blood flow in response to cognitive activity. We found that fNIRS was sensitive to variations in task difficulty in both real-life (flight simulator) and laboratory settings (tests measuring executive functions), showing increased concentration of oxygenated hemoglobin (HbO2) and decreased concentration of deoxygenated hemoglobin (HHb) in the prefrontal cortex as the tasks became more complex. Intensity of prefrontal activation (HbO2 concentration) was not clearly correlated to task performance. Rather, activation intensity shed insight on the level of mental effort, i.e., how hard an individual was working to accomplish a task. When combined with performance, fNIRS provided an estimate of the participants’ neural efficiency, and this efficiency was consistent across levels of difficulty of the same task. Overall, our data support the suitability of fNIRS to assess the mental effort related to human operations and represents a promising tool for the measurement of neural efficiency in other contexts such as training programs or the clinical setting.

## Introduction

Understanding the way the brain allocates mental resources according to the task demand is critically important for complex and high risk operational settings (e.g. piloting an aircraft, controlling air traffic, supervising a nuclear plant, etc.). The increase in mental workload in the face of a challenging task can lead to performance breakdown^1, 2^ with potentially fatal consequences. Measuring mental workload is complex as it represents the interplay between the demands of the environment (input load), human characteristics (capacities), and task performance (output). Thus, taking into account solely the task characteristics does now allow inferring the level of mental workload in an individual. There are many classical neuroimaging methods that allow measuring the neural substrates of mental workload in a continuous and unobtrusive way, such as electroencephalography (EEG)^3^, functional magnetic resonance imaging (fMRI)^4^, and positron emission tomography (PET)^5^. While these techniques have enabled an unprecedented window into the functioning of the human brain, they are not suited for use in ecological contexts. Indeed, EEG measures are subject to numerous artefacts due to head and/or body movements, and PET and fMRI require the subjects to lie supine and immobile during data acquisition. Therefore, there is a need for sensitive, continuous and robust measurements that are able to discriminate between various mental effort levels. Functional near infrared spectroscopy (fNIRS) is a relatively new and promising imaging technique that meets such measurement requirements, and the important advantage of being portable and field-deployable. This technique measures the oxygenated (HbO2) and deoxygenated (HHb) hemoglobin in the blood supply of the brain, and has been shown to discriminate between various mental effort levels (e.g. Ayaz *et al*.^6^). In contrast to the more classical neuroimaging techniques, fNIRS allows *in-vivo* imaging in ecological conditions with natural freedom of movement and in complex environments such as high-fidelity flight simulators.

The mental workload construct presupposes that task-related brain activity (e.g. perceptual, cognitive, and/or sensorimotor^7^) consumes a certain amount of mental resources - supposedly appreciable, multiple, independent, and limited^8^ - proportional to task difficulty. One method of measuring mental resource engagement is to quantify the energy consumption across several cellular levels of the brain to meet task demands^9^. The mobilisation of specialised neural pathways during cognitive activity relies on a continuous supply of oxygen and glucose through cerebral blood flow, mediated in particular by astrocyte-neuron metabolic cooperation^10, 11^. This change in blood flow due to neuronal activity is referred to as neurovascular coupling^12^ or functional hyperemia. Non-invasive functional brain imaging methods rely on this coupling to map brain activity, more specifically, the greater stimulus-induced focal augmentation of cerebral blood flow compared to the concomitant local increase in tissue metabolic rate^13^. This oversupply of oxygenated blood causes the fNIRS measurement of HbO2 to increase and the HHb to decrease^14^. Because the molecular mechanism at the basis of the neurovascular coupling is highly complex and may not necessarily be the same in all brain regions^15^, it would be unrealistic to assume that brain activity is linearly proportional to the hemodynamic response amplitude. Nevertheless, an accurate measurement of the neurovascular coupling with fNIRS can be a valuable neurophysiological marker for quantifying changes in brain activity in specific areas such as the prefrontal cortex. This can be achieved by linking the measure of the blood flow with the concept of mental workload, in line with previous work showing that mentally demanding tasks require resources in prefrontal-cortex-dependent functions^16–19^. Furthermore, it is well accepted that the frontal lobes are generally involved when tasks are complex, have novel demands or require considerable attention^20^. A good example of such a complex activity is piloting, as it takes place in a rapidly changing and uncertain environment, and has been shown to rely heavily on multiple prefrontal cortex-dependent higher order executive functions such as working memory, cognitive flexibility, or planning^21–23^. Not surprisingly, the few studies with fNIRS involving simulated^24, 25^ and real piloting scenarios^26^ converge to show increased oxygenation in the prefrontal cortex in response to cognitive demand.

So far, fNIRS technology has been used to estimate cognitive load in fundamental^27–29^, clinical^30^, aging^31, 32^, and human factors studies^6, 33^. For example, when using fNIRS to measure changes in activation during a standard *n*-back task, Ayaz *et al*.^6^ found consistent changes in oxygenation in the left dorsolateral prefrontal cortex as a function of memory load. This finding was further supported by an *n*-back study by Fishburn *et al*.^32^ showing linear increases in brain activation as a function of working memory load both in the right and the left prefrontal cortices. Variations in blood flow measured with fNIRS have also been associated with the engagement of executive functions such as mental flexibility^34^ or response inhibition^35^. When used in ecologically valid environments (e.g. piloting unmanned air vehicles), fNIRS has shown changes in oxygenation due to large increases in task difficulty; however, smaller differences in task difficulty could not be reliably differentiated (Ayaz *et al*., 2012, p.45). Moreover, several fNIRS studies have failed to establish a consistent and proportional relationships between mental workload and hemodynamic changes^36, 37^. A possible explanation is that participants sometimes disengage from the task, especially in more difficult levels exceeding their mental capability^37^.

Another aspect that remains to be explored is to what extent individual differences in the allocation of neural resources explain differences in task performance. While it is well-characterised in elderly people (e.g., see compensation hypothesis^38^), the study of the association between hemodynamic response and output performance in young healthy individuals is still in its infancy (in particular during ecologically valid tasks) and has thus far yielded mixed results^6, 39–41^ reported in the literature. It has been suggested that increased activation in the face of equal performances indicates less efficient neural processing^42^. The neural efficiency hypothesis of intelligence suggests that, for a given task and output performance, some individuals will need to allocate a substantial amount of mental resources, while others will reach the same results with much less mental effort^43, 44^. Thus, for the same output performance, two individuals may display different brain activities, and conversely, for the same brain activity, two individuals may display different output performances. The level of brain activation also seems to depend on task difficulty, since more intelligent individuals consume less energy when performing easy cognitive tasks (as assessed with fNIRS) but more energy when engaged in difficult mental operations^44^. A notable limitation of classical studies examining the neural efficiency hypothesis is that the tests themselves are not representatives of real-life settings (i.e., often performed in laboratory, similar to the contents of intelligence tests; see Neubauer & Fink^42^ for a review of the neuropsychological tests). It remains to be seen whether individuals who demonstrate high neural efficiency in laboratory cognitive tasks can demonstrate the same level of efficiency in real-world activities.

In order to build accurate prediction models of how the brain allocates resources in operational settings, we need to further understand how brain activity is related to performance. The objectives of the current study were two-fold. First, we aimed to contribute to the fNIRS validation literature by examining workload-related changes in brain activation in student pilots during performance of both simulated aircraft piloting tasks (natural, ecological context) and classical laboratory executive function tests involving spatial working memory and planning/reasoning (limited, laboratory context). Secondly, we aimed to explore cross-relationships between the hemodynamic response within the prefrontal cortex and performance in both scenarios.

There were three main hypotheses: 1) based on previous neuroimaging studies, we hypothesised that increasing difficulty during aircraft piloting and executive function tests (see material and methods section for details on the difficulty manipulation) would be both associated with increased HbO2 and decreased HHb, primarily in the dorsolateral prefrontal cortex; 2) according to the neural efficiency hypothesis that suggests that mental effort and output performance are not linearly associated, we hypothesised that individual prefrontal cortex activation should not be correlated with task performance; 3) finally, since the two aforementioned experimental settings recruit common cognitive functions (aircraft piloting involves executive functions such as working memory, cognitive flexibility or planning^21, 22^), we hypothesised that the neural efficiency index measured during the executive functions tests may correlate with the one for the piloting scenario.

## Results

### Changes in task performance and prefrontal activity with respect to task difficulty

#### Flight Simulator session

**Figure 1:**
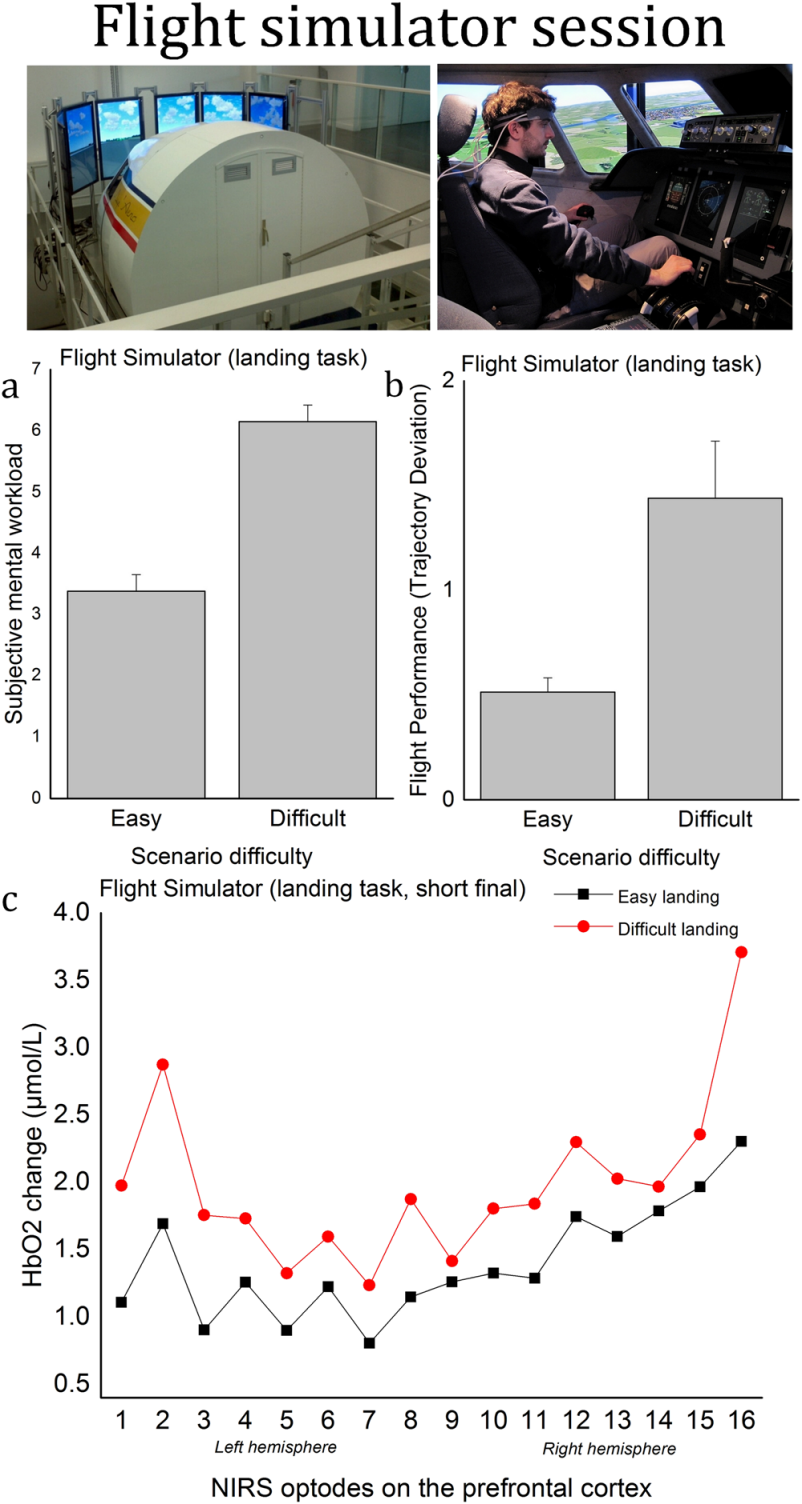
Flight simulator session. (**a**) Subjective mental workload during the easy and difficult landing scenario. Error bars represent the standard error of the mean; (**b**) trajectory deviations during the easy and difficult landing scenario. Error bars represent the standard error of the mean, smaller values indicate better performance. (**c**) Average HbO2 concentration changes (µmol/L) during the two flight scenarios (short final period) for the 16 optode locations.

*Perceived mental workload*. Since this landing exercise had not yet been previously validated in an experimental setting, a one-way (2 levels of difficulty) repeated measures ANOVA was used to compare the mental workload perceived by the participants during the two landing scenarios. Perceived mental workload was significantly higher in the difficult landing (*M* = 6.15, *SD* = 1.38) than in the easy landing (*M* = 3.38, *SD* = 1.38) (*F*(1, 25) = 73.72, *p* < 0.001, *η²*_*p*_ = 0.75), indicating that the scenario difficulty manipulation was successful (Fig. 1a).

#### Performance

A one-way (2 levels of difficulty) repeated measures ANOVA corroborated subjective results and showed that trajectory deviations were significantly higher during the difficult landing (*M* = 1.43, *SD* = 1.53) than during the easy landing scenario (*M* = 0.51, *SD* = 0.38,* F*(1, 25) = 12.69, *p* < 0.01, ${\eta }_{p}^{2}$ = 0.34), see Fig. 1b.

#### Prefrontal activity

For HbO2, a three-way (2 levels of difficulty × 16 optode locations × 2 periods of time) repeated measures ANOVA showed a significant main effect of the period of time (*F*(1, 25) = 115.85, *p* < 0.001, ${\eta }_{p}^{2}$ = 0.82). The HbO2 concentration was higher during the short final (late phase of the landing in which the control of the aircraft is more complex due to the proximity of the airfield) than during the final approach (early phase of the landing). There was a main effect of the optode location (*F*(15, 375) = 9.19, *p* < 0.001, ${\eta }_{p}^{2}$ = 0.27). In particular, optode #16, in the area of the right dorsolateral prefrontal cortex (DLPFC), demonstrated a higher concentration change of HbO2 than all other optodes except for #2/#15 (*p* < 0.05 in all significant comparisons). There was also a period of time × difficulty interaction (*F*(1, 25) = 9.05, *p* < 0.01, ${\eta }_{p}^{2}$ = 0.27), showing an effect of the difficulty only during the short final (*p* < 0.05), see Fig. 1c. This latter result is consistent with the flying convention since the task difficulty primarily occurs when the airfield is close. For HHb, the three-way (2 levels of difficulty × 16 optode locations × 2 periods of time) repeated measures ANOVA showed a significant main effect of the period of time (*F*(1, 25) = 143.84, *p* < 0.001, ${\eta }_{p}^{2}$ = 0.85), with lower HHb concentrations during the short final than during the final. There was also a main effect of the optode location (*F*(15, 375) = 5.41, *p* < 0.001, *η²*_*p*_ = 0.18), in particular, HHb concentration in the optodes #15/#16, located in the right part of the prefrontal cortex, was lower than in 9 other optodes, namely #1/#3–10 (*p* < 0.05 in all significant comparisons). There was also a significant optode location × period of time (*F*(15, 375) = 3.23, *p* < 0.001, ${\eta }_{p}^{2}$ = 0.11). The ANOVA did not reveal any significant effect of the difficulty *(p* > 0.05).

### Executive functions session - Spatial working memory (SWM)

**Figure 2:**
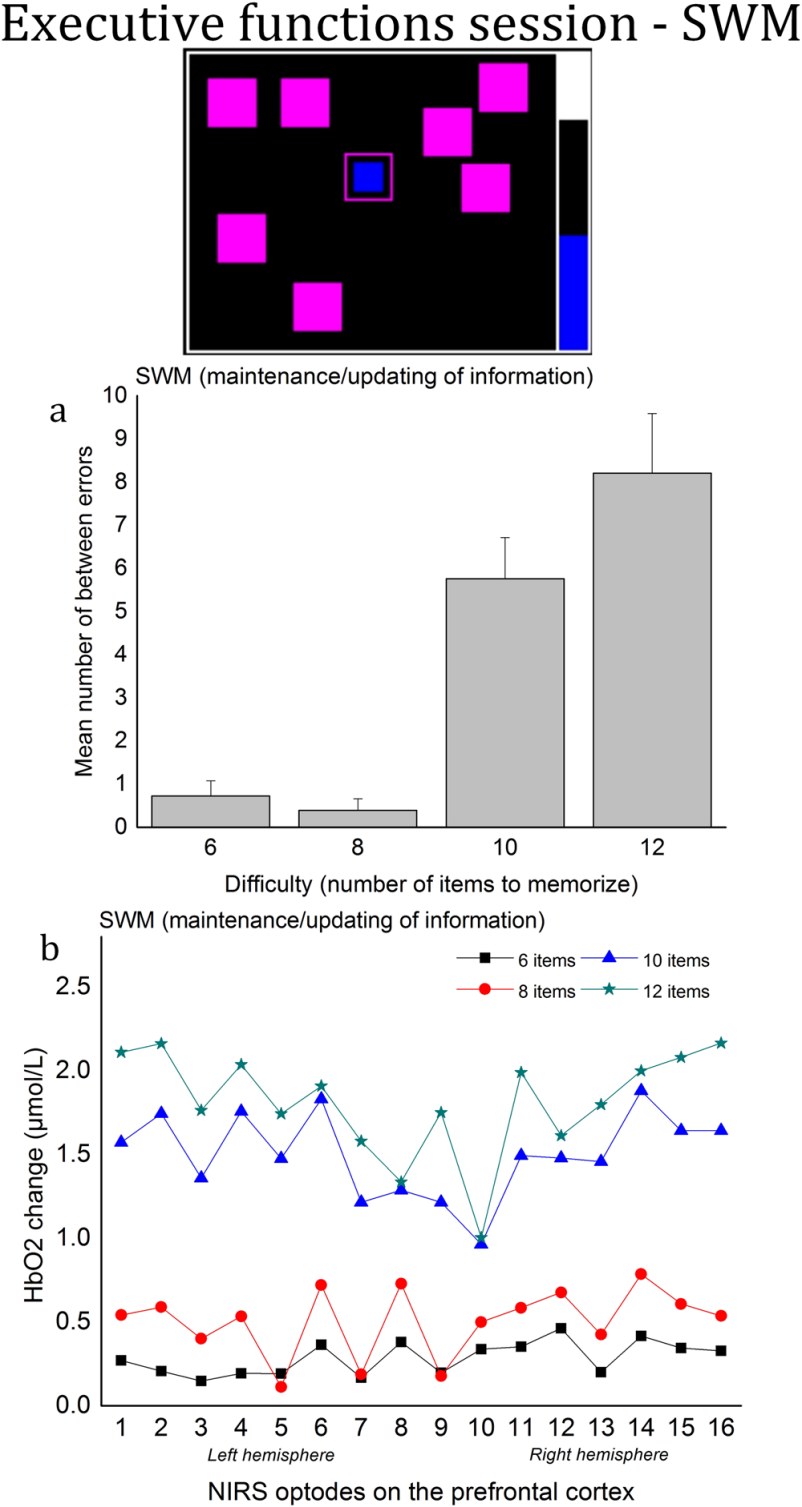
Executive functions session – Spatial Working memory (SWM). (**a**) Mean number of errors across the four levels of difficulty. Error bars represent the standard error of the mean. (**b**) Average HbO2 concentration changes (µmol/L) across the four levels of difficulty for the 16 optode locations.

*Performance*. A one-way (4 levels of difficulty) repeated measures ANOVA was used to determine the effect of task difficulty on spatial working memory performance. The analysis showed that performances were significantly affected by the level of difficuly, *F*(3, 51) = 24.84, *p* < 0.001, ${\eta }_{p}^{2}$ = 0.59, see Fig. 2a. Post hoc testing showed an increased number of errors with 12 items (*M* = 8.20, *SD* = 5.85) versus 6/8 items (*M* = 0.72, *SD* = 1.48; *M* = 0.38, *SD* = 1.14, respectively; *p* < 0.001 in both comparisons) and with 10 items (*M* = 5.75, *SD* = 4.03) versus 6/8 items (*p* < 0.001 in both comparisons).

#### Prefrontal activity

For HbO2, a two-way (4 levels of difficulty × 16 optode locations) repeated measures ANOVA showed that the HbO2 concentration increased with difficulty, *F*(3, 48) = 37.77, *p* < 0.001, ${\eta }_{p}^{2}$ = 0.70, see Fig. 2b. Post hoc testing showed a higher HbO2 concentration with 12 items versus 6/8 items (*p* < 0.001 in both comparisons) and a higher HbO2 concentration with 10 items versus 6/8 items (*p* < 0.001 in both comparisons). The ANOVA revealed a significant effect of the optode location, *F*(15, 240) = 1.79, *p* < 0.05, ${\eta }_{p}^{2}$ = 0.10, and a difficulty × optode location interaction, *F*(45, 720) = 2.78, *p* < 0.001, ${\eta }_{p}^{2}$ = 0.15. For HHb, the two-way (4 levels of difficulty × 16 optode locations) repeated measures ANOVA showed a main effect of the difficulty level, with HHb concentration decreasing with difficulty, *F*(3, 48) = 9.96, *p* < 0.001, ${\eta }_{p}^{2}$ = 0.38. The HHb concentration was lower with 12 items versus 6/8 items (*p* < 0.01 in both comparisons) as well as with 10 items versus 6 items (*p* < 0.01). Finally, there was a significant difficulty × optode location, *F*(45, 720) = 1.84, *p* < 0.001, ${\eta }_{p}^{2}$ = 0.10).

### Executive functions session - One Touch Stockings (OTS)

**Figure 3:**
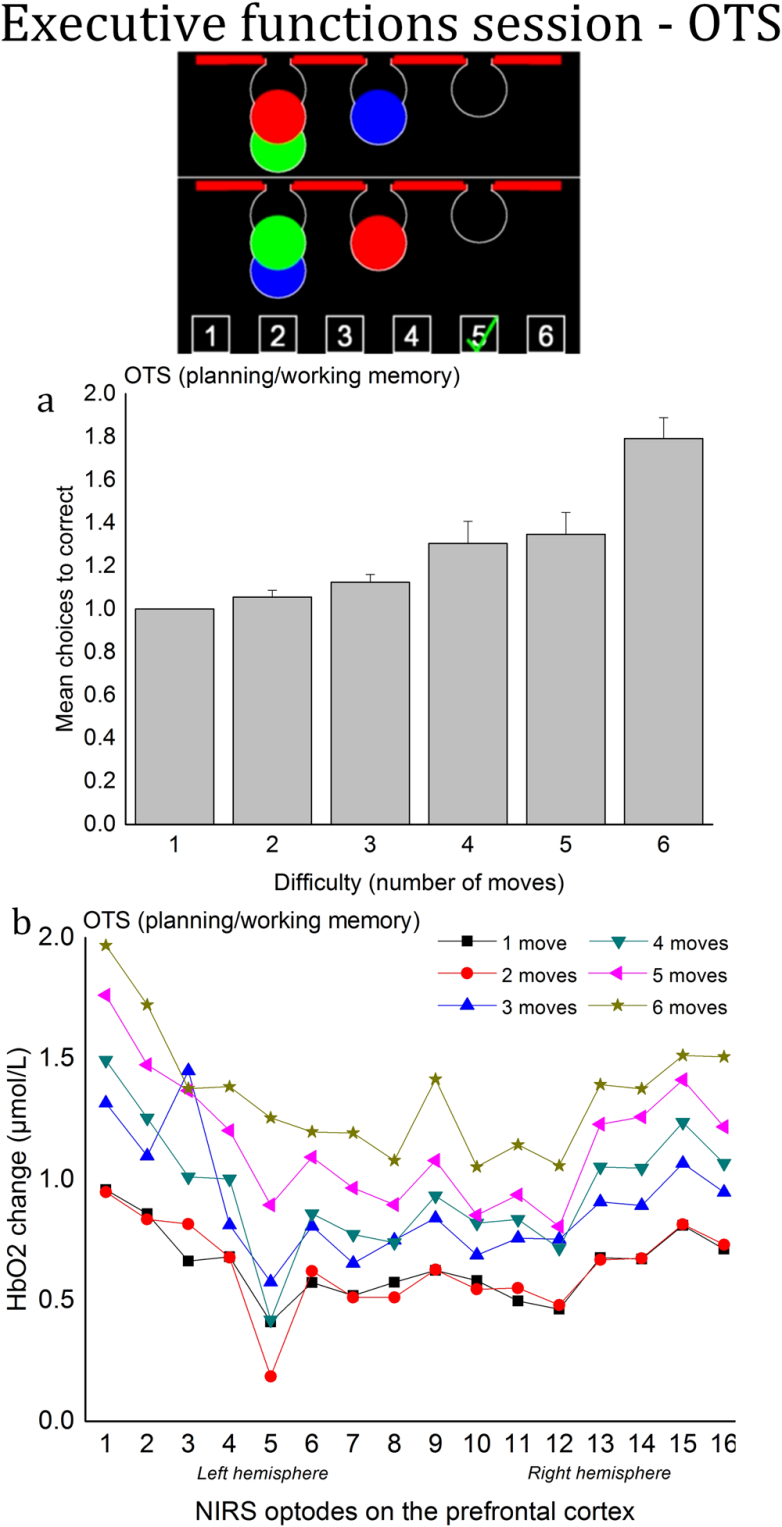
Executive functions session - One Touch Stockings (OTS). (**a**) Mean number of errors across the six levels of difficulty. Error bars represent the standard error of the mean. (**b**) Average HbO2 concentration changes (µmol/L) across the six levels of difficulty for the 16 optode locations.

*Performance*. A one-way (6 levels of difficulty) repeated measures ANOVA was used to test the effect of OTS task difficulty on the spatial planning and reasoning performance, namely the number of errors prior to correct choice. As expected, the analysis showed that the mean number of erroneous choices was significantly affected by the level of difficulty, *F*(5, 85) = 17.31, *p* < 0.001, ${\eta }_{p}^{2}$ = 0.50, see Fig. 3a. Post hoc testing revealed an increased number of errors with 6 moves (*M* = 1.79, *SD* = 0.41) versus all other difficulties (*M* = 1.00, *SD* = 0.00; *M* = 1.05, *SD* = 0.13; *M* = 1.12, *SD* = 0.15; *M* = 1.30, *SD* = 0.43; *M* = 1.34, *SD* = 0.42, in ascending order; *p* < 0.001 in all comparisons), with 5 moves versus 1/2 moves (*p* < 0.001 and *p* < 0.05, respectively), and with 4 moves versus 1 move (*p* < 0.05).

#### Prefrontal activity

For HbO2, a two-way (6 levels of difficulty × 16 optode locations) repeated measures ANOVA showed that the HbO2 concentration increased with difficulty, *F*(5, 85) = 18.18, *p* < 0.001, ${\eta }_{p}^{2}$ = 0.52, see Fig. 3b. Post hoc testing showed a higher HbO2 concentration with 6 moves versus 1/2/3/4 moves (*p* < 0.001 in all comparisons), a higher HbO2 concentration with 5 moves versus 1/2 moves (*p* < 0.001 in both comparisons), and a higher HbO2 concentration with 4 moves versus 1/2 moves (*p* < 0.05 in both comparisons). There was also a significant effect of the optode location, *F*(15, 255) = 1.71, *p* < 0.05, ${\eta }_{p}^{2}$ = 0.09, with HbO2 concentration in optode #5 being lower than optode #1.

For HHb, the two-way (6 levels of difficulty × 16 optode locations) repeated measures ANOVA revealed that the HHb concentration decreased with difficulty, *F*(5, 85) = 6.32, *p* < 0.001, ${\eta }_{p}^{2}$ = 0.27. Post hoc testing showed a lower HHb concentration with 6 moves versus 1/2 moves (*p* < 0.01 in both comparisons) and a lower HHb concentration with 5 moves versus 1/2 moves (*p* < 0.01 in both comparisons). Finally, there was also an effect of the optode location, *F*(15, 255) = 1.79, *p* < 0.05, ${\eta }_{p}^{2}$ = 0.10. In particular, the HHb concentration was lower in optode #16 than in optode #5.

#### Prefrontal activity during the three tasks

**Figure 4:**
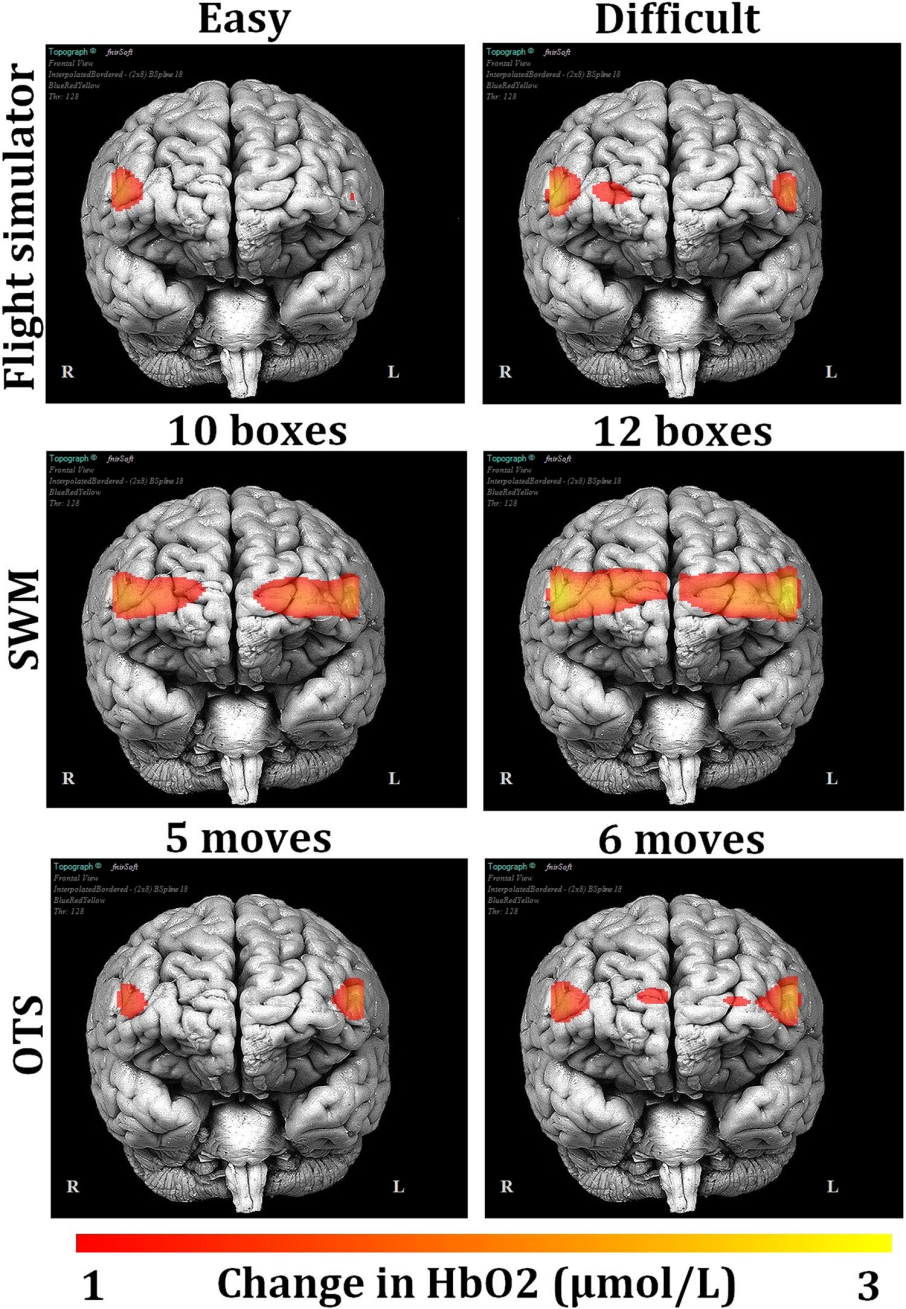
Topographical map of the change in HbO2 with respect to the two levels of difficulty during the flight simulator session (easy/difficult landing) or the two highest levels of difficulty during SWM (10/12 boxes) and OTS (5/6 moves).

An additional two-way (3 tasks × 2 levels of difficulty) repeated measures ANOVA (all optodes averaged) considering the two levels (flight simulator, easy/difficult landing) or the two highest levels (SWM, 10/12 boxes; OTS, 5/6 moves) of difficulty of the neuropsychological tests confirmed increased HbO2 concentration as the task became more complex, *F*(1, 17) = 4.28, *p* < 0.05, *η*²_*p*_ = 0.21. The ANOVA also revealed a main effect of the task, *F*(2, 34) = 4.18, *p* < 0.05, *η*²_*p*_ = 0.20, with SWM provoking higher HbO2 concentrations than the flight simulator (*p* < 0.05), see Fig. 4. However, it must be noted that this analysis was performed on the HbO2 concentration changes averaged across the whole duration of the flight simulator scenarios. As shown in the supplementary material (Tables S2 and S3), there was a peak in HbO2 concentration change coinciding with the last phase of the difficult landing scenario. This peak was greater than that observed during the highest level of difficulty of SWM (1.98 µmol/L and 1.81 µmol/L, respectively).

### Correlation between piloting and executive functions test performances

A supplementary correlation analysis revealed that task performance achieved during the two highest levels of difficulty of SWM and OTS was not correlated with the flying performance (for either landing scenario) in both landings (*r* < 0.40 in all comparisons).

### The relationship between prefrontal cortex activity and task performance

Eighteen correlation (3 tasks × 2 levels of difficulty × 3 prefrontal regions) analyses were conducted to assess the association between HbO2 changes over the prefrontal cortex (HbO2 concentration averaged on 3 regions of the prefrontal cortex, rescaled to [0, 1] interval with the formula, $x^{\prime} =\frac{x-\,{\rm{\min }}(x)}{{\rm{\max }}(x)-\,{\rm{\min }}(x)}$, where min and max stand for the lowest and highest individual values in the sample, respectively) and task performance (inversed and rescaled to [0, 1] interval with the following formula, $x^{\prime} =\frac{{\rm{\max }}(x)-x}{{\rm{\max }}(x)-\,{\rm{\min }}(x)}$, so that smaller initial value, i.e. good performance, would correspond to 1) during the two landing scenarios and each of the two highest levels of difficulty of the two laboratory tasks. To simplify this analysis, the averaged HbO2 concentration on three areas of interest − left prefrontal (optodes 1–6), anterior prefrontal (optodes 7–10), and right prefrontal (optodes 11–16) – were used in lieu of a series of correlations at each optode location.

The analysis showed significant correlations in only one test, with a moderate negative correlation between the performance in OTS at the 5 moves difficulty level and the HbO2 concentration in the anterior prefrontal cortex (*r*(18) = −0.52). The higher the right prefrontal cortex was activated, the lower the performance was (the participants made more attempts to find the correct solution). None of the other correlations were significant (*r* < 0.40 in all cases).

### Neural efficiency during the flight simulator task and the neuropsychological tests

Building on the previous analysis, a neural efficiency index was calculated (assuming that HbO2 variations were predominantly influenced by the neuronal activity) for each task (flight simulator, SWM, OTS) and its respective two levels (easy/difficult landing) or two highest levels (SWM, 10/12 boxes; OTS, 5/6 moves) of difficulty for each participant. This index was defined as the inverted and rescaled performance subtracted from the rescaled HbO2 concentration (both metrics were calculated in the previous section), with higher index values [interval: 0, 1] indicating higher neural efficiency.

**Figure 5:**
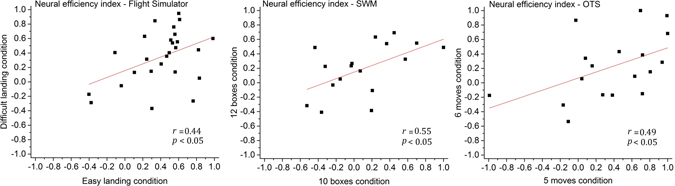
From left to right, intra-task correlations between the individual neural efficiency indexes attained during the easy and difficult landing scenarios and the two highest levels of difficulty of SWM and OTS. The figure shows the prefrontal region in which the correlations were significant (right prefrontal cortex).

Correlations between the easy and difficult landing scenarios and between the two highest levels of difficulty of SWM and OTS were first conducted to determine whether the neural efficiency index was consistent intra-task. Intra-task neural efficiency index was systematically correlated in all tasks, namely between the easy and difficult landing scenarios in the right prefrontal area (*r*(26) = 0.43); between 10 and 12 boxes in SWM in the right prefrontal area (*r*(17) = 0.54) and between 5 and 6 moves in OTS also in the right prefrontal area (*r*(18) = 0.49), see Fig. 5. No significant correlation between the neural efficiency index attained during the executive tasks and during the flight simulator session was found (*r* < 0.40).

## Discussion

In this study, we used fNIRS to monitor the prefrontal activity of student pilots performing two landing scenarios (easy and difficult) in a realistic flight simulator and two neuropsychological tests. The main goals of this research were to contribute to the ongoing validation of the sensitivity of fNIRS measurements to various mental effort levels, and to better understand how variation in prefrontal cortex activity correlates with task performance. Furthermore, we examined whether individuals who demonstrate a high neural efficiency index while performing the laboratory cognitive tasks also attain a high neural efficiency index in the realistic flight simulation settings.

Regarding the first hypothesis, the results confirmed that increased task difficulty in both laboratory setting and flight simulator setting was associated with degraded performance, higher self-reported measurements of mental workload (measured only during the landing scenarios), as well as HbO2 increase and HHb decrease in the prefrontal cortex. These outcomes agree with previous studies reporting increased oxygenation as a function of task difficulty in a variety of scenarios such as simulated aircraft landing^24^, video games^45^, air traffic control^6^, and laboratory tasks such as the *n*-back^28, 46^. We also found that the right dorsolateral prefrontal cortex was predominantly recruited during the landing scenario, which confirms the important role of this brain region during realistic settings of high mental workload^6^. This dominance of the right DLPFC during the landing task can also indicate possible enhanced mental stress and attentional effort associated with the aviation task. Indeed, such right hemisphere dominance during stress-inducing tasks^47–49^ or during tasks requiring higher level of vigilance^50^ have been already reported in previous literature. The comparison of HbO2 concentration among the three tasks revealed that laboratory tasks may elicit equivalent or even higher fNIRS activity than more ecological tasks such as the flight simulator. However, there was a greater peak in HbO2 concentration during the last phase of the difficult landing in comparison to that observed during the highest level of difficulty of the tests.

As mentioned in a recent paper by Tachtsidis and Scholkmann^14^, fNIRS hemodynamic responses can be modulated by various phenomena unrelated to neurovascular coupling, thus producing changes in systemic variables and leading to non-neuronal driven changes in hemodynamics/oxygenation (i.e. intracerebral hemodynamics caused by task related systemic activity and/or extracerebral hemodynamics associated for example with changes in heart rate or blood pressure). Classical filtering methods such as the band-pass filter employed in this study are generally sufficient for removing this non-task related activities, like the low-frequency oscillation that arises from fluctuations in the blood flow and hemoglobin oxygenation at a global circulatory system level^51^. Even if these systemic influences cannot be completely excluded, the fact that HHb concentration variation showed an opposite pattern than HbO2 (HHb concentrations decreased with difficulty) supports the idea that variations in oxygenated hemoglobin were mainly brain-related (as evidenced in supplementary material, see Supplementary Figures S1, S2 and S3). Moreover, we did not introduce variations of stress, discomfort or movements across the different levels of task difficulty, which could have provoked large task-related systemic changes and thus interfere with the brain functional response. For example, walking or grasping has been shown to reduce or increase HbO2 concentration in the prefrontal region^37, 52^. In order to better control these possible confounding factors, future studies may include the recording of autonomic nervous measurements like heart rate and peripheral oxygen saturation, as this could help to further correct for systemic influences in the cerebral NIRS signal^14^.

On the whole, our findings demonstrated the sensitivity of fNIRS to detect variation in mental effort in both ecological and laboratory environments. This technique may be a strong alternative to current in-field techniques such as a continuous questioning of the operator’s subjective mental workload, which may create additional workload or diminish the realism of the task. *In-situ* monitoring of pilots’ mental effort can be of great interest for providing feedback to the operator himself or to the automated system with which he is interacting. The introduction of intelligent adaptive systems that can adjust the mental workload by taking charge of a wide variety of tasks^53, 54^ while letting the operator focus on high level tasks is a relevant application of the real-time monitoring of the cerebral activity^55^. Indeed, it would be useful for the system to detect hazardous situations where the operator is mentally vulnerable, in order to engage more automated tasks to compensate for potential operator failure or shortcomings. fNIRS can also have applications for the evaluation and certification of human machine interfaces, as it would allow an objective assessment of the mental effort, for example, in operators as they interact with technology. Elements of the technology that generate excessive workload and poor performance should be redesigned as to avoid forcing unnecessary user adaptation. Additionally, providing this objective feedback to the human-machine interface would allow the system to adapt its level of automation according to the current mental workload.

Regarding the second hypothesis, the analysis of the association between averaged prefrontal HbO2 concentration and simulator/laboratory performance showed mixed results with a negative correlation in only one task (during the OTS task at the 5 moves difficulty level only). Such a dissociation between performance and brain activity was also found by other authors using fNIRS^6, 56^ and it indicates that mental effort (as measured by HbO2 concentration change) is not necessarily linked to task performance. First, there may be significant differences in the neural efficiency level of the participants, hence explaining the lack of significant correlation between performance and HbO2 concentration. The neural efficiency hypothesis of intelligence states that intelligent individuals display lower brain activation while performing cognitive tasks (see the review by Neubauer and Fink^42^). For a given output performance, some individuals need to use a substantial amount of resources, while more intelligent individuals will obtain the equivalent result with much less effort (i.e. higher efficiency). This hypothesis has been confirmed in various intellectual activities such as reading^43^, working memory^57^, and executive tasks^58^. Secondly, previous findings highlight that expertise also has a significant impact on behavioural performance and neural efficiency in domain-specific tasks^59^. This effect appears to be largely independent of the level of intelligence^60^. The study by McKendrick, Ayaz, Olmstead, and Parasuraman^61^ revealed that training was accompanied by negative correlations between verbal span performance and the hemodynamic response in both frontal lobes, suggesting an increase in processing efficiency^42, 62^. This study demonstrated that, even if our sample consisted of student pilots, there may be differences in how individuals have internalised their flying skills (some of them had prior experience with light aircraft or flight simulators). Indeed, with practice and learning, skills become more automated and less associated with prefrontal cortical recruitment (e.g., see Toni, I., Krams, M., Turner, R. and Passingham^63^). Consequently, good flying performance can be associated with lower HbO2 concentration for those individuals with more flight experience as well as with higher HbO2 concentration for those who have less experience but are still able to perform the task. Thirdly, the test durations were quite long, which can increase variability in the hemodynamic signal. For example, in the Wagner *et al*.^39^ study that showed that prefrontal and temporal activations predicted recall of verbal experiences, the hemodynamic response was observed during a short period of time (a few seconds) whereas some of our trials lasted several tens of seconds (e.g. 10/12 boxes during SWM). Other authors with long task durations also failed to find significant correlations between cerebral activity and performance, for example, during video gaming sessions with children and adults^56^. Overall, given the potential inter-individual variations in neural efficiency and/or level of expertise, brain activation in itself may not be a relevant marker of task performance, in particular in complex and ecological tasks.

In contrary to our third hypothesis, the neural efficiency index attained during the laboratory tests was not conclusively correlated to the one attained during piloting. Of course, the cognitive functions in the laboratory tests did not cover the full spectrum of executive functions required in the flight simulator tasks. This suggestion is supported by the lack of correlation between the performance on the executive tests and the one in the flight scenario. Piloting is a complex task that engages a variety of cognitive functions in parallel^22, 64^. Therefore, it is possible that while the spatial working memory, and planning and reasoning executive functions were in use in our landing scenario, these specific executive functions were overshadowed by another, or a combination of other, cognitive function(s) not measured by the tests. The low variability of participants’ cognitive performance may also have contributed to this null-result (all participants were *ab initio* pilots that were previously selected due to their high intellectual capacities).

In contrast, we found that the neural efficiency index was consistent within a given cognitive domain. Indeed, the individual neural efficiency index obtained within two levels (easy/difficult landing) or two higher levels (SWM, 10/12 boxes; OTS, 5/6 moves) of difficulty were systematically correlated, illustrating that individuals’ processing efficiency may be predicted across tasks if they engage very similar cognitive functions.

## Conclusion

The present study highlights the potential use of the fNIRS technology to provide non-obtrusive data sampling to assess mental effort in both ecological and laboratory tasks. Increased task difficulty in both settings was systematically associated with increases in HbO2 and decreases in HHb concentrations in the prefrontal cortex. In SWM and OTS, the increased number of errors in the highest levels of difficulty was particularly consistent with the rise of the HbO2 concentration. For example, the HbO2 concentration change was close to 0 in the easiest conditions (6 and 8 moves) in SWM, in which the error rate was low, but HbO2 concentration rose drastically in addition to the number of errors in the two highest levels of difficulty (10 and 12 boxes). In OTS, the rise of errors was more monotonic, consistent with the HbO2 concentration change. Relevant applications of using fNIRS to estimate mental effort encompass real-time human operator monitoring^25^, characterisation of the impact of training programs^6^, and human machine interface evaluation^65^.

Despite the fact that group level analysis revealed a concomitant increase of trajectory deviation/error and HbO2 concentration in the most difficult conditions, our results also confirmed that predicting individual behavioural performance in complex tasks on the sole basis of the measure of the fNIRS activity can be unrealistic most of the time. For the same performance, two individuals may display important differences in prefrontal activation, because a higher domain-specific neural efficiency allows a more effective use of the specialised brain area. Neural efficiency can be also increased with expertise as individuals develop appropriate and efficient strategies with practice. Thus, inter-individual variations in intelligence and expertise limit the possibility to find linear correlations between brain activation and output performance. Future studies may compare pilots with different levels of flying experience in order to better investigate the effects of training and practice in such a complex environment.

We believe that the measurement of the mental effort with fNIRS, considered jointly with behavioural performance, represents a reliable estimate of the participant’s neural efficiency. Such a variable, not visible through observable behaviour, gives a credible indicator as to how hard the brain is working to reach a given performance. This indicator may have applications in the evaluation of patients with subtle cognitive defects (e.g. adults with mild cognitive impairment^66^) barely detectable by behavioural observations, through the accurate monitoring of the neural efficiency. Finally, we show that individual processing efficiency was consistent across tasks only when they shared very similar cognitive functions. Is this sense, the neural efficiency index measured in the executive function tests did not correlate with the neural efficiency index measured in the flight simulator. The generalisation of the results obtained in laboratory to real-life ecological conditions should be made with caution, and further studies involving other tests must be conducted. The neuroergonomics approach is a great opportunity to assess the external/ecological validity of experiments conducted in controlled situations (high internal validity).

## Material and Methods

### Participants

Twenty-six young student pilots (*élèves pilotes de ligne*, EPL) from the Ecole Nationale de l’Aviation Civile, i.e. the national civil aviation school in France (ENAC; Toulouse, France; mean age: 20.6, *SD* = 1.1, two females), were recruited to participate in the two sessions: the flight simulator task and then the laboratory executive function tests. All participants were novices on the specific simulator used in this study. However, their experience with light aircraft or flight simulators varied (mean flying experience was 53 hours, ranging from 0 to 450). All participants gave written informed consent in accordance with the local ethical board committee. The study complied with the Declaration of Helsinki for human experimentation and was approved by the medical Committee (CPP du Sud-Ouest et Outre-Mer IV, n°CPP15-010b/2015-A00458-41). The participant visible in Fig. 1 gave their informed consent for the publication of identifying images.

### Flight simulator session

Two landing scenarios of different cognitive demands (easy and difficult) were performed at Blagnac airport (Toulouse, France). The order in which the landing was performed was counterbalanced across participants. The initial conditions for both landing scenarios were identical: altitude of 2500 feet, heading of 143 degrees, speed of 130 knots, starting 6 miles from the airfield threshold. In both experimental scenarios, the instrument landing system (ILS) was available to help perform the approach. In the easy landing scenario, the external visibility was Ceiling And Visibility OK (CAVOK, i.e. perfect) and there was no crosswind. In the difficult landing scenario, there was no external visibility (dense cloud layer) above 100 feet of altitude (dense cloud layer) and there was a strong crosswind. In the latter scenario, the use of the ILS was mandatory due to the low visibility. The difficult landing condition was intended to generate a higher mental effort than the easy one. Raw flight performance was defined as the root mean square error flying performance (distance between the performed flight trajectory, for the vertical and horizontal axis, and the ideal landing trajectory given by the ILS). The duration of each landing scenario was similar, approximately 2.5–3 minutes.

### Executive functions session

The tasks were chosen from the Cambridge Neuropsychological Automated Test Battery (CANTAB) framework. These tests and their variants have been used in a variety of applications and have been validated by several brain imaging techniques, such as EEG, fMRI, and PET.

#### Spatial Working Memory test

The SWM test is designed to recruit and assess the ability to maintain and update spatial information in working memory^67^. The goal of the task is to find tokens which are hidden one at a time within a random arrangement of boxes. Once a token has been found within a box, the participant does not need to inspect the same box again, as another token would never yet again appear there. Chase, Clark, Sahakian, Bullmore, and Robbins^68^ showed that performance during the training phase was correlated with the amount of damage to the DLPFC (Brodmann Area 46/9) in a group of patients with frontal lesions. After practice trials with 3 boxes, assessed trials were administered with increasing difficulty (6, 8, 10 and 12 boxes). Administration time was approximately 8 minutes. Performance was measured in terms of the mean number of errors within each level of task difficulty (i.e. number of boxes), defined as the number of times the participant revisited a box in which a token has previously been found.

#### One Touch Stockings

The OTS test evokes spatial planning abilities. For each trial, a lower and an upper set of balls were shown in pockets. Participants had to mentally find the minimum number of moves it would take to make the lower set match the upper set. The balls were akin to billiard balls resting in different-sized sockets (one-, two-, or three-ball capacity); they are stackable and cannot be removed from a pocket if another ball rests on top of it. The DLPFC is generally engaged during this task as shown by similar paradigms in fMRI^69–71^, PET^72, 73^ and transcranial magnetic stimulation^74^. After four practice trials, 24 assessed trials requiring 1 to 6 moves were administered in a pseudo-random order, with 6 moves trials being administered starting from the 16^th^ trials. In case of an erroneous answer, participants had to try again. Administration time was approximately 8 minutes. The performance measurement was the mean number of erroneous choices before reaching the correct response.

### fNIRS recording and data analysis

**Figure 6:**
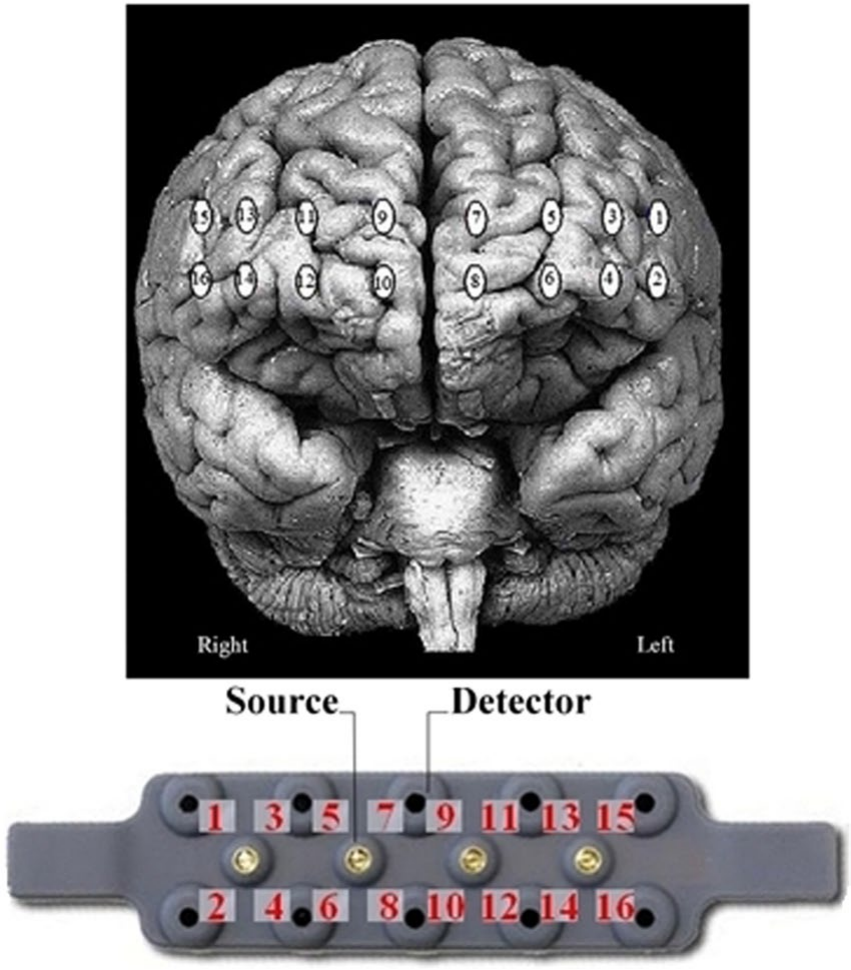
Up: optode locations on a normalised brain template. Down: fNIR100 headband and associated optode locations numbering.

During the entire duration of each landing scenario and each neuropsychological test, the hemodynamics of the prefrontal cortex was recorded using the fNIR100 stand-alone functional brain imaging system (Biopac™, see Fig. 6). Sixteen optodes recorded the hemodynamics at a frequency of 2 Hz with a 2.5 cm source-detector separation.

COBI Studio software version 1.2.0.111 (Biopac™ systems) was used for data acquisition and visualisation, and the fNIRS raw data were pre-processed using fnirSoft version 1.3.2.3 (Biopac™ systems). For each participant, the variations in light absorption at two different peak wavelengths (730 nm and 850 nm) were used to calculate changes of HbO2 and HHb concentrations (both in µmol/L) using the modified Beer–Lambert Law (MBLL). Before each flight scenario and each executive task, participants were asked to relax for approximately two minutes, and a ten-second baseline measurement was then performed. Changes in HbO2 and HHb concentration from this ten-second rest period baseline^2, 6, 75^ were computed over the entire time course of each task. To remove long-term drift^76^, higher-frequency cardiac or respiratory activity and other noise with other frequencies than the target signal^77–80^, we used a band-pass FIR filter with an order of 20 (0.02–0.40 Hz) on this raw time series of HbO2 and HHb signal changes. After this process, a correlation-based signal improvement (CBSI^76, 81^) algorithm was used to filter out spikes and to improve signal quality based on the assumed negative correlation between HbO2 and HHb^82^. The data was then visually inspected and one participant with a readily visible saturated signal in all optodes was excluded from the SWM fNIRS analysis (Supplementary Figures S1, S2 and S3 show individual filtered signal changes in concentrations of both HbO2 and HHb during the easy and difficult landing and during SWM and OTS, respectively). HbO2 and HHb concentrations were then averaged across all trials for each condition. Optodes with clearly abnormal concentrations (mean concentration values lower than −15 µmol/L or higher than +15 µmol/L) were excluded. More specifically, we excluded 1 channel for one participant during the landing session and 3 channels for another participant during the laboratory session. Missing values were substituted by the participant’s mean concentration calculated on all other available optodes during the condition.

ANOVA was used to test the hypothesis as to whether increasing difficulty during the tasks (piloting, laboratory) would result in decreased task performance and changes in HbO2 and HHb (separate ANOVAs were conducted for each signal). Since the final moment of the approach (when the aircraft is near the runway threshold) is known to potentially generate an important increase of the workload, we also studied the effect of the period of time on HbO2 and HHb concentration (final = aircraft above 1000 feet; short final = aircraft below 1000 feet). Post hoc testing was conducted with Tukey’s Honestly Significant Difference (Tukey HSD). A series of Bravais-Pearson correlations between the piloting and laboratory task performances and respective changes in HBO2 were used to test the second hypothesis as to whether there was a significant relationship between prefrontal cortex activity and task performance. Lastly, the third hypothesis on the inter-task relationship was also tested using a series of Bravais-Pearson correlations between the neural efficiency indexes calculated for each task. In order to reduce the number of analyses, the second and the third hypothesis were tested on HbO2 signal only. HbO2 is considered as the most sensitive parameter of activity-dependent changes in regional cerebral blood flow in optical measurements studies^83–85^, and several other studies highlighted that HbO2 is particularly sensitive to mental workload variations^2, 25^. All statistical analyses were performed using Statistica 7.1 (StatSoft ©) and significance was defined at α = 0.05. Bravais-Pearson correlations were interpreted according to the absolute value of *r*_s_, with “moderate” (0.40 ≤ *r* ≤ 0.59), and “strong” (0.60 ≤ *r* ≤ 0.79) correlation strengths. Correlations with *r* < 0.40 were considered non-significant. Supplementary Table S1 shows the ANOVA summary table of the effects of the independent variables on HbO2 and HHb during the flight simulator task and the two neuropsychological tests. Supplementary Table S2 shows the mean and standard deviations for HbO2 and HHb changes in the whole prefrontal cortex (16 voxels averaged) according to the level of difficulty during the flight simulator session. Similar data is provided for SWM and OTS in Supplementary Tables S3 and S4 respectively.

### Procedure

During the flight simulator session, participants used the A300 flight simulator (PEGASE), located at ISAE-Supaero (Toulouse, France). The simulator reproduces angular acceleration along three axes (roll, pitch, and altitude). Participants sat in the captain’s seat (front-left) of the simulator. They were instructed to perform two landing scenarios during which their performance and brain activity would be recorded. Before the two experimental landing scenarios, each participant underwent training consisting of two similar landings: one with external visibility and no crosswind (similar to the easy landing), another also with external visibility but with a moderate crosswind (similar to the difficult landing except that the wind was weaker in this training scenario). This exercise allowed the participants to gain familiarity with the flight simulator and contributed to reduce a possible learning effect. After these two training scenarios, participants were equipped with the fNIR100 stand-alone functional brain imaging system (Biopac™) and they performed the two experimental assessment landing scenarios during which their prefrontal activity was recorded. Immediately after each experimental landing scenario, participants completed a subjective mental workload evaluation on a 1–7 scale. This simplified procedure has shown to be significantly correlated with the NASA Task Load Index (TLX) questionnaire^86^. This flight simulator session lasted approximately 1 hour.

**Figure 7:**
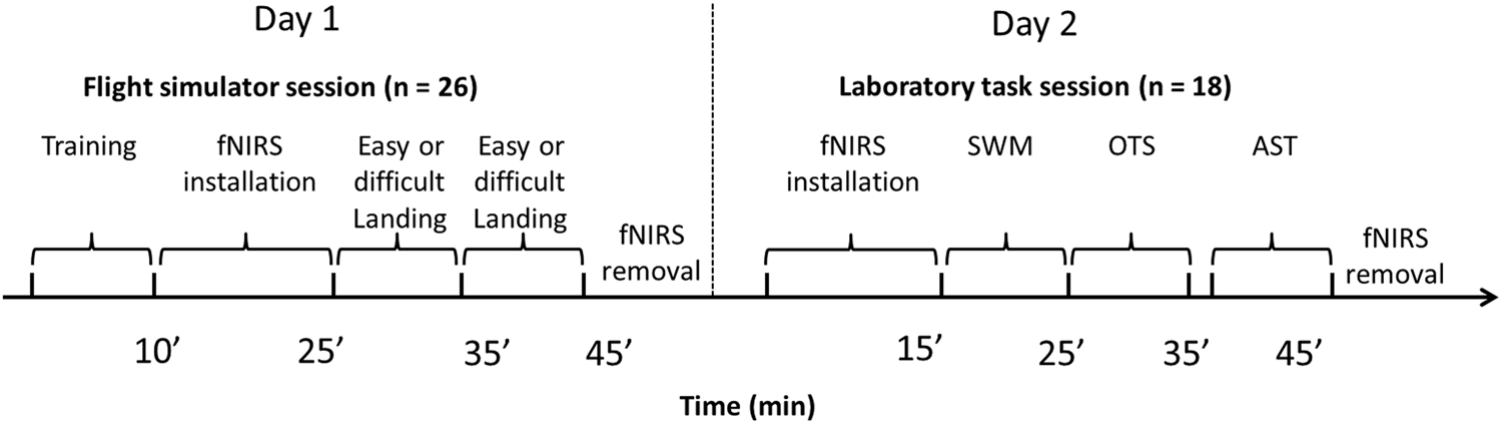
Timeline of experimental protocol for each session. The time interval between the flight simulator and the executive functions sessions was approximately two months.

From the original 26 participants that performed the flight simulator session, 18 participants returned for the executive functions test session which was held approximately two months later. This session took place in a quiet laboratory room with constant and dimmed light. Participants sat in front of a tablet (Motion Computing J3600/J3500 i3 equipped with windows 7) that housed the neurocognitive testing software, and they were equipped with the fNIR100 system. Instructions were given before each test. Multiple practice trials preceded the assessed trials for each test (see Fig. 7 for the procedure timeline). A third test (AST) was performed by the participants but this is the subject of a subsequent manuscript. The executive functions session also lasted for approximately 1 hour.

## Electronic supplementary material

Supplementary Information
