# Author Correction: Mental workload and neural efficiency quantified in the prefrontal cortex using fNIRS

**DOI:** 10.1038/s41598-018-25614-2

**Published:** 2018-05-02

**Authors:** Mickaël Causse, Zarrin Chua, Vsevolod Peysakhovich, Natalia Del Campo, Nadine Matton

**Affiliations:** 1 Institut Supérieur de l’Aéronautique et de l’Espace (ISAE-SUPAERO), Toulouse, France; 2 Ecole de psychologie, Université Laval, Québec, Canada; 3 Centre of Excellence in Neurodegeneration of Toulouse, NeuroToul, CHU, Toulouse, France; 4 Toulouse NeuroImaging Center, ToNIC, University of Toulouse, Inserm, UPS, Toulouse, France; 5 University of Cambridge, Department of Psychiatry, Addenbrooke’s Hospital, Cambridge, UK; 6 Ecole Nationale de l’Aviation Civile, Toulouse, 31055 France; 7 Laboratoire CLLE-LTC, 5 Allée Antonio Machado, 31100 Toulouse, France

Correction to: *Scientific Reports* 10.1038/s41598-017-05378-x, published online 12 July 2017

This Article contains a typographical error.

In the Results section,

“This index was defined as the inverted and rescaled performance subtracted from the rescaled HbO2 concentration (both metrics were calculated in the previous section), with higher index values [interval: 0, 1] indicating higher neural efficiency.”

should read:

“This index was defined as the inverted and rescaled performance minus the rescaled HbO2 concentration (both metrics were calculated in the previous section), with higher index values [interval: -1, 1] indicating higher neural efficiency.”

