# Supplementary material for: Mental workload and neural efficiency quantified in the prefrontal cortex using fNIRS
Source: Sci Rep. 2017 Jul 12;7:5222. doi: 10.1038/s41598-017-05378-x (PMC5507990; doi:10.1038/s41598-017-05378-x)
Supplement: Supplementary file 1 — Supplementary Information [file 41598_2017_5378_MOESM1_ESM.doc]

**Mental workload and neural efficiency quantified in the prefrontal cortex using fNIRS**

**Supplementary Information**

Mickaël Causse, Zarrin Chua, Vsevolod Peysakhovich, Natalia Del Campo, and Nadine Matton

**Supplementary Figures**


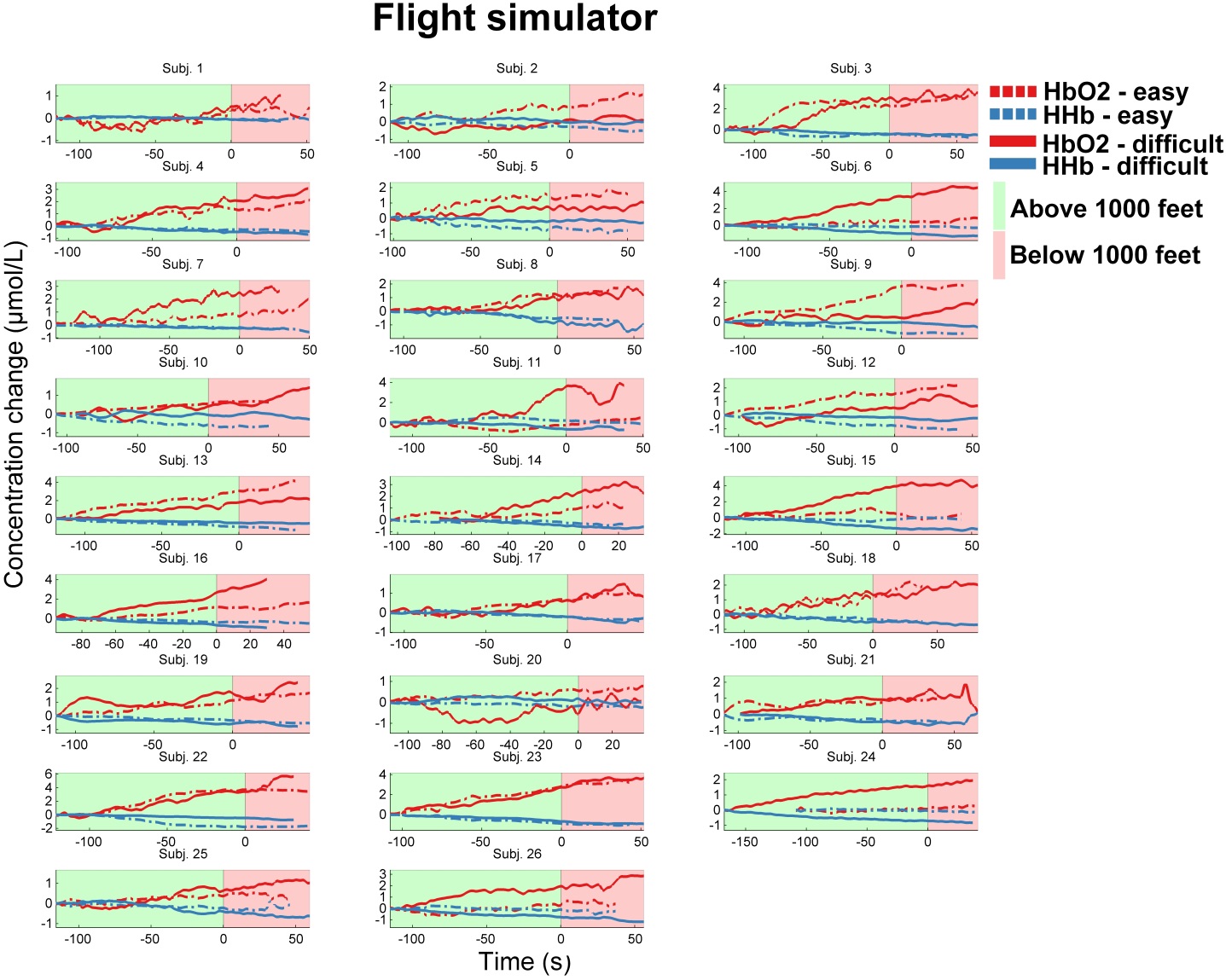


Supplementary Figure S1. Individual concentration changes in HHb and HbO2 during the easy and difficult landings. Time of the two landings is aligned to the beginning of the short final (aircraft went below 1000 feet). The displayed signal was band-pass filtered (0.02Hz-0.40Hz) and submitted to the CBSI method.


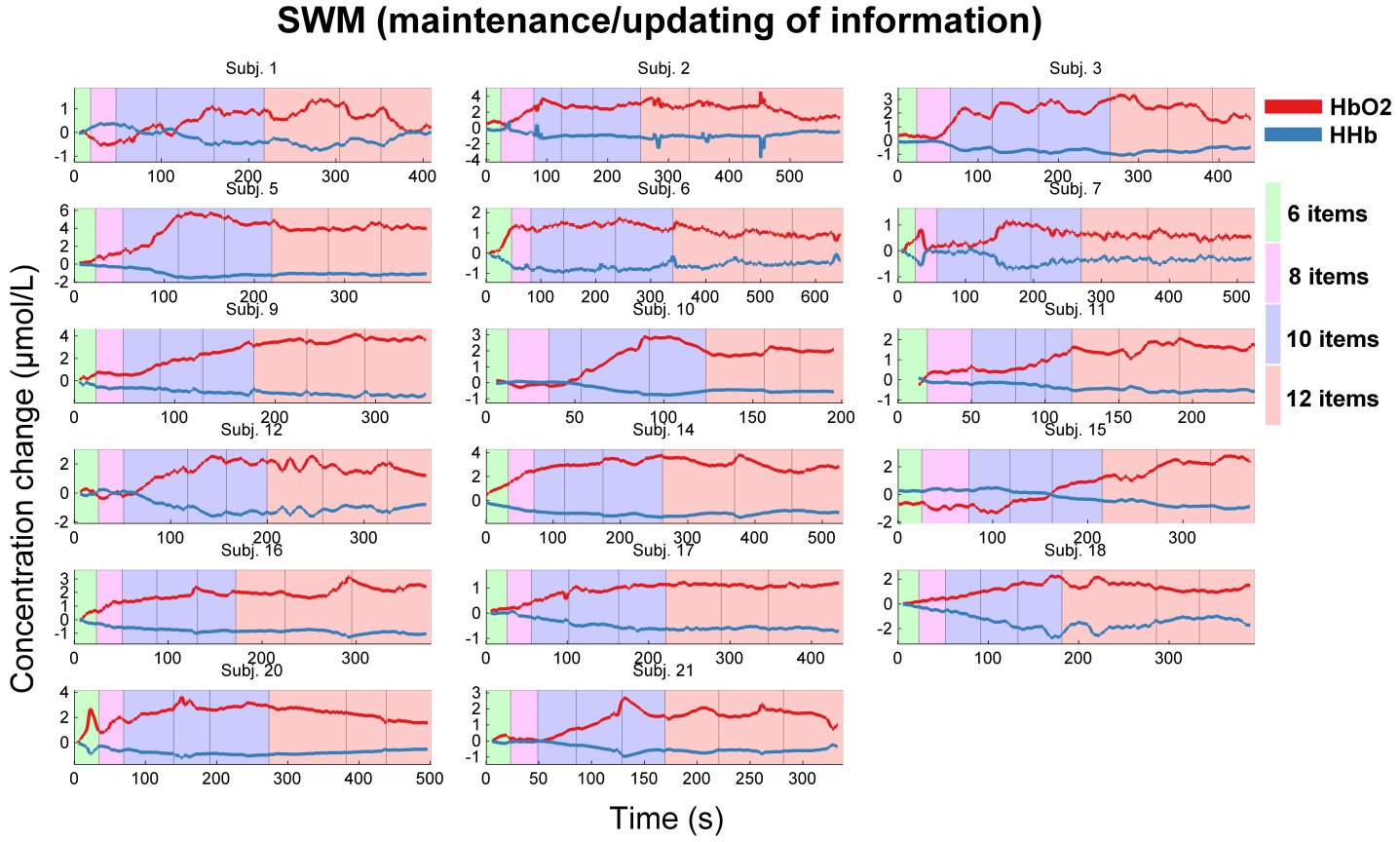


Supplementary Figure S2. Individual concentration changes in HHb and HbO2 during the Spatial Working Memory (SWM) task. The displayed signal was band-pass filtered (0.02Hz-0.40Hz) and submitted to the CBSI method.


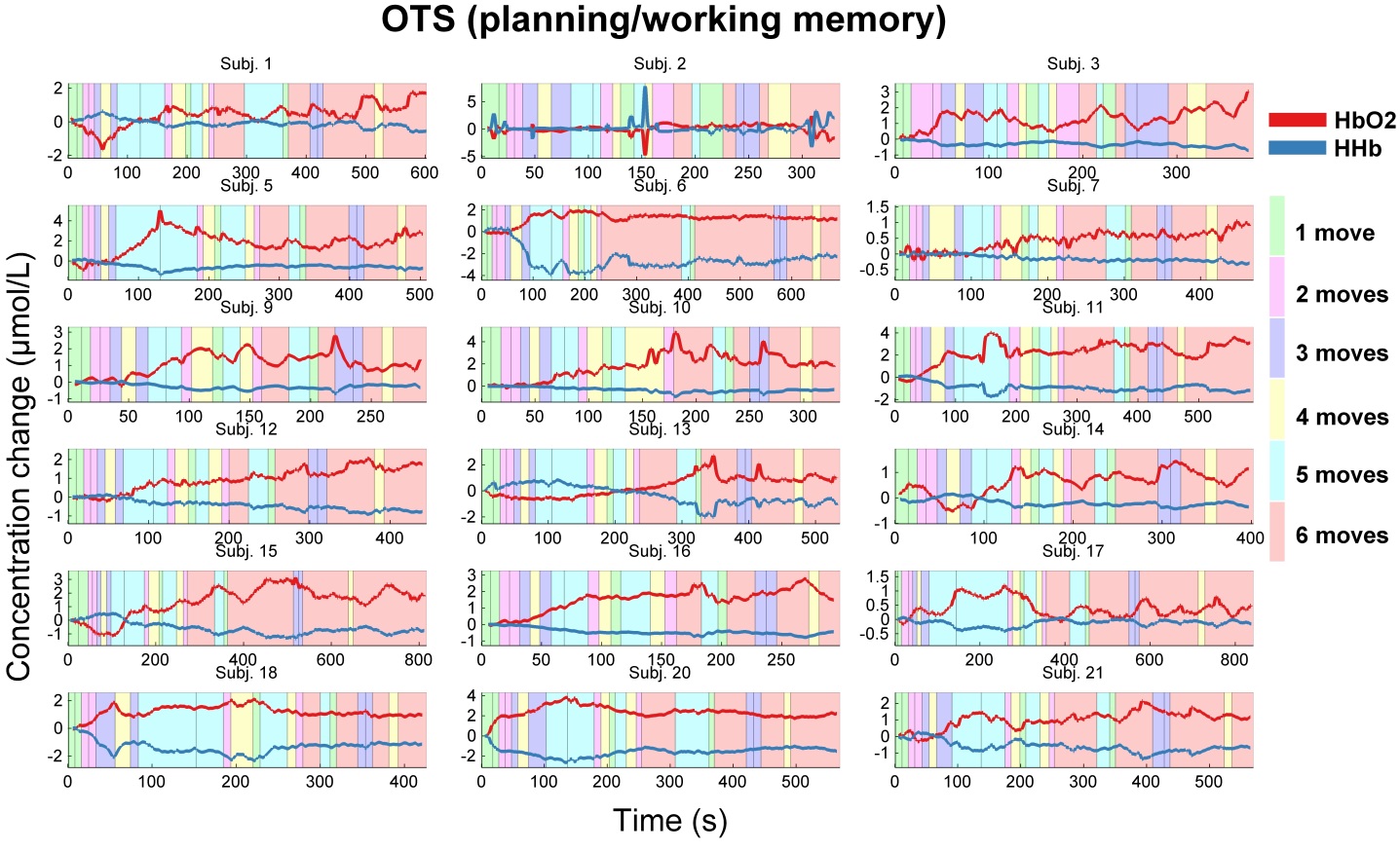


Supplementary Figure S3. Individual concentration changes in HHb and HbO2 during the One Touch Stockings of Cambridge (OTS) task. The displayed signal was band-pass filtered (0.02Hz-0.40Hz) and submitted to the CBSI method.

**Supplementary Table**

Supplementary Table S1. ANOVA summary table showing the effects of the independent variable on HbO2 and HHb during the flight simulator and the two neuropsychological tests (SWM, OTS). MS = mean square; df = degree of freedom. ”*”represents statistical significance at *p* < 0.05,“**”represents statistical significance at *p* < 0.01 and “***”represents statistical significance at *p* < 0.001.

|  | | Variables | MS | *F* | df | *p* | *η²_p_* |
| --- | --- | --- | --- | --- | --- | --- | --- |
| **Flight simulator** | |  |  |  |  |  |  |
|  | HbO2 |  |  |  |  |  |  |
|  |  | Difficulty | 47.34 | 2.36 | 1, 25 | 0.13 | .07 |
|  | | Time*** | 437.08 | 115.85 | 1, 25 | *< 0.001* | .82 |
|  | | Optode *** | 17.02 | 9.19 | 15, 375 | *< 0.001* | *.27* |
|  | | Difficulty × Time** | 27.03 | 9.05 | 1, 25 | *< 0.01* | *.27* |
|  | | Difficulty × Optode | 1.58 | 1.68 | 15, 375 | *0.051* | *.06* |
|  | | Time × Optode *** | 1.10 | 3.20 | 15, 375 | *<0 .001* | *.11* |
|  | | Difficulty × Time × Optode * | 0.29 | 1.75 | 15, 375 | *<0 .05* | *.07* |
|  | HHb |  |  |  |  |  |  |
|  |  | Difficulty | 0.02 | 0.00 | 1, 25 | 0.93 | *.00* |
|  | | Time*** | 40.00 | 143.84 | 1, 25 | *< 0.001* | *.85* |
|  | | Optode *** | 1.43 | 5.41 | 15, 375 | *< 0.001* | *.18* |
|  | | Difficulty × Time | 0.59 | 1.64 | 1, 25 | 0.21 | *.06* |
|  | | Difficulty × Optode | 0.14 | 0.93 | 15, 375 | 0.52 | *.04* |
|  | | Time × Optode *** | 0.13 | 3.23 | 15, 375 | *< 0.001* | *.11* |
|  | | Difficulty × Time × Optode | 0.019 | 1.03 | 15, 375 | 0.41 | *.04* |
| **SWM** | |  |  |  |  |  |  |
|  | HbO2 |  |  |  |  |  |  |
|  |  | Difficulty*** | 176.13 | 37.77 | 3, 48 | *< 0.001* | *.70* |
|  | | Optode * | 1.96 | 1.79 | 15, 240 | *< 0.05* | *.10* |
|  | | Difficulty × Optode *** | 0.55 | 2.78 | 45, 720 | *< 0.001* | *.15* |
|  | HHb |  |  |  |  |  |  |
|  |  | Difficulty*** | 20.26 | 9.96 | 3, 48 | *< 0.001* | *.38* |
|  | | Optode | 1.73 | 1.12 | 15, 240 | *0.33* | *.07* |
|  |  | Difficulty × Optode *** | 0.28 | 1.84 | 45, 720 | *< 0.001* | *.10* |
| **OTS** | |  |  |  |  |  |  |
|  | HbO2 |  |  |  |  |  |  |
|  |  | Difficulty*** | 22.90 | 18.18 | 5, 85 | *< 0.001* | *.52* |
|  |  | Optode * | 4.698 | 1.71 | 15, 255 | *< 0.05* | *.09* |
|  | | Difficulty × Optode | 0.16 | 0.90 | 75, 1275 | 0.70 | *.05* |
|  | HHb |  |  |  |  |  |  |
|  |  | Difficulty*** | 6.05 | 6.32 | 5, 85 | *< 0.001* | *.27* |
|  | | Optode * | 5.24 | 1.79 | 15, 255 | *< 0.05* | *.10* |
|  | | Difficulty × Optode | 0.23 | 0.92 | 75, 1275 | 0.65 | *.05* |

Supplementary Table S2. Means and standard deviations for HbO2 and HHb changes in the whole prefrontal cortex (16 voxels averaged) according to the level of difficulty during the flight simulator session.

| **Flight simulator** | | Easy | |  | Difficult | |
| --- | --- | --- | --- | --- | --- | --- |
|  | | *M* | *SD* |  | *M* | *SD* |
| HbO2 | |  |  |  |  |  |
|  | Final | 0.63 | 0.92 |  | 0.70 | 0.94 |
|  | Short final | 1.39 | 1.43 |  | 1.98 | 1.84 |
| HHb | |  |  |  |  |  |
|  | Final | -0.22 | 0.36 |  | -0.19 | 0.29 |
|  | Short final | -0.49 | 0.57 |  | -0.53 | 0.56 |

Supplementary Table S3. Means and standard deviations for HbO2 and HHb changes in the whole prefrontal cortex (16 voxels averaged) according to the level of difficulty during Spatial Working Memory (SWM) test performance.

| **SWM** | | 6 items | |  | 8 items | |  | 10 items | |  | 12 items | |
| --- | --- | --- | --- | --- | --- | --- | --- | --- | --- | --- | --- | --- |
|  | | *M* | *SD* |  | *M* | *SD* |  | *M* | *SD* |  | *M* | *SD* |
| HbO2 | |  |  |  |  |  |  |  |  |  |  |  |
|  |  | 0.28 | 0.51 |  | 0.50 | 0.98 |  | 1.50 | 1.25 |  | 1.81 | 1.28 |
| HHb | |  |  |  |  |  |  |  |  |  |  |  |
|  |  | -0.14 | 0.46 |  | -0.30 | 1.19 |  | -0.61 | 0.94 |  | -0.75 | 0.75 |

Supplementary Table S4. Means and standard deviations for HbO2 and HHb changes in the whole prefrontal cortex (16 voxels averaged) according to the level of difficulty during One Touch Stockings (OTS) test performance.

| **OTS** | | 1 move | |  | 2 moves | |  | 3 moves | |  | 4 moves | |  | 5 moves | |  | 6 moves | |
| --- | --- | --- | --- | --- | --- | --- | --- | --- | --- | --- | --- | --- | --- | --- | --- | --- | --- | --- |
|  | | *M* | *SD* |  | *M* | *SD* |  | *M* | *SD* |  | *M* | *SD* |  | *M* | *SD* |  | *M* | *SD* |
| HbO2 | |  |  |  |  |  |  |  |  |  |  |  |  |  |  |  |  |  |
|  |  | 0.64 | 0.68 |  | 0.63 | 0.88 |  | 0.89 | 0.99 |  | 0.95 | 1.14 |  | 1.15 | 1.13 |  | 1.35 | 1.08 |
| HHb | |  |  |  |  |  |  |  |  |  |  |  |  |  |  |  |  |  |
|  |  | -0.32 | 0.71 |  | -0.30 | 0.95 |  | -0.49 | 1.07 |  | -0.46 | 1.31 |  | -0.61 | 1.14 |  | -0.66 | 0.91 |
